# What Is a Homœopathic Prescription?*Read at the Minnesota Homœopathic State Institute in May, 1895.

**Published:** 1895-09

**Authors:** G. E. Clark

**Affiliations:** Stillwater, Minn.


### WHAT IS A HOMŒOPATHIC PRESCRIPTION?*

G. E. Clark, M. D., Stillwater, Minn.

Much has been said about the imperfections of our materia medica and of the crying necessity for immediate and radical revision of the voluminous material at hand. While it is doubtless true that imperfections still exist, and always will remain in any materia medica compiled by human hands, it is manifestly incorrect to charge upon the materia medica faults that arise from an incorrect *use* of this most useful book. Rather, we think, the great and crying need of the homœopathic profession at present is—not more, or a revised ma-

---

* Read at the Minnesota Homœopathic State Institute in May, 1895.

teria medica but *a more accurate and scientific use of that we have.*

Our present compilation of drug provings is probably as nearly perfect as it is reasonable to expect.

More drugs will be added and others better defined; but the good work has been going on, and, it is to be hoped, will continue. So that something will always remain to which we are yet to attain. Moreover, compared with other text-books on medical subjects, we think the homœopathic materia medica will stand a most excellent comparison in point of accurate and scientific investigation and arrangement.

In point of painstaking and indefatigable labor it stands a living monument to the giant intellects that composed it.

Is it not true that much of the revision that is at present demanded is to fit an inaccurate and unhomœopathic use of a homœopathic materia medica?

Trite as this subject may be, I have presumed upon your patience to ask the plain question, "What is a homœopathic prescription?" I do so because we do well to keep constantly in mind some of the underlying principles of our divine art. Our right to exist as physicians depends on our ability to cure the sick. Our claim to the title homœopathic lies in our ability to prove this the superior method of healing—*the Law of Cure.*

I. The first answer to this question will be in the form of a negation in the statement that *A homœopathic prescription is not necessarily a prescription made by a homœopathic physician.*

*The Organon*, in conformity to which we claim the title homœopathic, lays down definite rules for our guidance. A prescription is only a homœopathic prescription when made in accordance with these fundamental principles. It is, alas! too true and much to be regretted by suffering humanity, at least, that many a prescription and the sign over the office-door have so little affinity. It is in no censorious spirit that I speak these words. It is a fact well known that the homœopathic profession is suffering sorely at the hands of men who claim the title homœopathic, yet have not the faintest right or title to the use of that word. They know not and comprehend not any of the deeper truths of our beneficent law. Our enemies speak of them as shining examples of the futility of our peculiar practice. In justice to ourselves and to those who look to us for relief in the quickest and safest manner the meaningless use of this word should be dropped, and *only* used when applied to those who both *understand* and *use* the principle of Similia.

II. A second negation is: *That a homœopathic prescription does not consist in any particular medicine or form of medicine, pills, vials, potencies, or specifics.*

This statement may seem even more commonplace than the first. It is, nevertheless, true that many physicians, without ever having read *The Organon* and scouting many, if not all, of its essential principles, still flatter themselves with the delusion that they are proper homœopathic physicians because they use homœopathic remedies or give "small pills." Well could it be said of them, as of old, "Depart; I never knew you"! Such never realize the beneficent results of *pure* Homœopathy nor credit its wondrous results.

At the present time, with great *eclat*, the virtues of certain homœopathic "specifics" are heralded to the world. They are a snare and a delusion. People are deceived thereby. Push the button, and out comes the needed homœopathic remedy. People count this homœopathic treatment. Ye gods! How low in the dust has our noble art fallen if by such practice we are to be judged. Hahnemann was thoroughly alive to the importance of a clear knowledge of *what Homœopathy is.* Over and over again he impressed upon his followers the statement that Homœopathy does not consist in forms that shift with the notions of the patient or whims of the physician, but rather in *principles*—in a definite law as fixed and unchanging as are the laws of nature. That law is expressed in the relation of *drug and disease symptoms.* These disease symptoms do not consist in an assumed pathological state that is to be extracted from the interior of the system or driven out by lurid incantations, but rather, as defined in § 19 of *The Organon,* as *aberrations of normal healthy function.* In the preceding section it states,

*other* than these expressions of the disturbed normal function, there is *no* guide to the remedial cure of the affection.

This is a vital point in our law of cure. For therapeutic purposes we *must* possess a *full* and *complete* statement of the various aberrations of normal function—the *Totality of the Symptoms.* There is no other guide to the certain selection of a curative remedy.

This dictum of the master was no passing whim, which we can with advantage lightly set aside. To *know* the possibilities of Homœopathy—to know it as it really is, we must accept the statement that, without first obtaining a perfect picture of the disease symptoms, we will not be able to perfectly adapt the known drug symptoms. The knowledge obtained in the first process is absolutely essential to the perfect realization of the second. This knowledge of the disease symptoms can only be obtained by an orderly and systematic procedure.

Hahnemann considered this the most important as well as difficult part of the therapeutic process. Hence, in § 84 *et seq.*, he gave minute instructions for obtaining and recording exactly and exhaustively the history and peculiarities of the case presenting for professional care. Herein lies the genius of a homœopathic prescription :

First in the possession of accurate and complete knowledge of the external manifestations of the disturbed vital function.

By external manifestations, I mean such positive knowledge of actual facts as may be obtained by the senses of sight, hearing, and touch. Speculative or unproven theories as to possible internal conditions can have little value in point of accuracy to the prescriber.

Second. The selection of drugs where pathogenetic effects are most similar to those sought to be removed by the therapeutic process. This consummated, and *only* then, have we as physicians produced a homœopathic prescription.

How infinitely superior is this careful and orderly procedure to the hasty *guess* work of other methods, much too frequently indulged in. But best of all, and commensurate to the fidelity with which the master's instructions are carried out, is the superiority of the *results accomplished*—results that are not possible by less accurate and systematic methods.

